# Radiomics predictive modeling from dual-time-point FDG PET *K*_*i*_ parametric maps: application to chemotherapy response in lymphoma

**DOI:** 10.1186/s13550-023-01022-0

**Published:** 2023-07-26

**Authors:** Rezvan Samimi, Isaac Shiri, Yashar Ahmadyar, Jörg van den Hoff, Alireza Kamali-Asl, Alireza Rezaee, Fereshteh Yousefirizi, Parham Geramifar, Arman Rahmim

**Affiliations:** 1 Department of Medical Radiation Engineering, Shahid Beheshti University, Tehran, Iran; 2 Division of Nuclear Medicine and Molecular Imaging, Geneva University Hospital, 1211 Geneva 4, Switzerland; 3 PET Center, Institute of Radiopharmaceutical Cancer Research, Helmholtz-Zentrum Dresden-Rossendorf, 01328 Dresden, Germany; 4 Department of Nuclear Medicine, University Hospital Carl Gustav Carus, Technische Universität Dresden, 01307 Dresden, Germany; 5 Khatam PET/CT Center, Khatam Hospital, Tehran, Iran; 6 Department of Integrative Oncology, BC Cancer Research Institute, Vancouver, BC Canada; 7 Research Center for Nuclear Medicine, Tehran University of Medical Sciences, Tehran, Iran; 8 Departments of Radiology and Physics, University of British Columbia, Vancouver, BC Canada

**Keywords:** Dynamic PET, Radiomics, Artificial intelligence, Lymphoma, Predictive model, Engineered radiomics

## Abstract

**Background:**

To investigate the use of dynamic radiomics features derived from dual-time-point (DTP-feature) [^18^F]FDG PET metabolic uptake rate *K*_*i*_ parametric maps to develop a predictive model for response to chemotherapy in lymphoma patients.

**Methods:**

We analyzed 126 lesions from 45 lymphoma patients (responding *n* = 75 and non-responding *n* = 51) treated with chemotherapy from two different centers. Static and DTP radiomics features were extracted from baseline static PET images and DTP *K*_*i*_ parametric maps. Spearman’s rank correlations were calculated between static and DTP features to identify features with potential additional information. We first employed univariate analysis to determine correlations between individual features, and subsequently utilized multivariate analysis to derive predictive models utilizing DTP and static radiomics features before and after ComBat harmonization. For multivariate modeling, we utilized both the minimum redundancy maximum relevance feature selection technique and the XGBoost classifier. To evaluate our model, we partitioned the patient datasets into training/validation and testing sets using an 80/20% split. Different metrics for classification including area under the curve (AUC), sensitivity (SEN), specificity (SPE), and accuracy (ACC) were reported in test sets.

**Results:**

Via Spearman’s rank correlations, there was negligible to moderate correlation between 32 out of 65 DTP features and some static features (*ρ* < 0.7); all the other 33 features showed high correlations (*ρ* ≥ 0.7). In univariate modeling, no significant difference between AUC of DTP and static features was observed. GLRLM_RLNU from static features demonstrated a strong correlation (AUC = 0.75, *p* value = 0.0001, *q* value = 0.0007) with therapy response. The most predictive DTP features were GLCM_Energy, GLCM_Entropy, and Uniformity, each with AUC = 0.73, *p* value = 0.0001, and *q* value < 0.0005. In multivariate analysis, the mean ranges of AUCs increased following harmonization. Use of harmonization plus combining DTP and static features was shown to provide significantly improved predictions (AUC = 0.97 ± 0.02, accuracy = 0.89 ± 0.05, sensitivity = 0.92 ± 0.09, and specificity = 0.88 ± 0.05). All models depicted significant performance in terms of AUC, ACC, SEN, and SPE (*p* < 0.05, Mann–Whitney test).

**Conclusions:**

Our results demonstrate significant value in harmonization of radiomics features as well as combining DTP and static radiomics models for predicting response to chemotherapy in lymphoma patients.

**Supplementary Information:**

The online version contains supplementary material available at 10.1186/s13550-023-01022-0.

## Introduction

In clinical oncology, medical imaging technologies have evolved from simple diagnostic tools to a source of valuable clinical information over the years [1, 2]. In addition, the emergence of new technologies and the requirements of precision medicine has given rise to a promising field of radiomics [3, 4]. Radiomics is an image data-mining framework that makes it possible to extract a variety of quantitative imaging features from medical images and identify potential relationships with clinical and biological findings. As a result, radiomics may increase the precision of diagnosis, prediction, and prognosis to improve clinical decision-making for many diseases, including lymphoma [1, 5–12].

[^18^F]FDG or other PET radiopharmaceutical uptake patterns within a tumor have been characterized by identifying imaging features (intensity, heterogeneity, and shape) reflecting biological characteristics, such as cellular density, proliferation rate, hypoxia, necrosis, and angiogenesis [13, 14]. Several attempts have been made to evaluate the relationship between quantitative parameters of [^18^F]FDG uptake and the treatment response of lymphoma [15–23]. Parvez et al*.* [16] found that metabolic tumor volume (MTV) correlates with response to therapy in a retrospective study of 82 aggressive B-cell lymphoma patients. However, MTV represents the total volume of tumor activity and does not reflect spatial distribution, heterogeneity, and shape of lesions. Lue et al. [17] and Tatsumi et al. [18] reported that the radiomics features of [^18^F]FDG PET promise predictive values for treatment response in patients with Hodgkin and follicular lymphoma, respectively. In a retrospective study of 30 patients, Sun et al. [24] found that the standardized uptake value (SUV), the MTV, some texture features, and the tumor location were useful parameters in interim response prediction of primary gastrointestinal diffuse large B-cell lymphoma (DLBCL).

Based on the literature, it remains to be established how important different biomarkers are for predicting outcomes in lymphoma. For instance, in diffuse large B-cell lymphoma, Adams et al. [25] discovered that the national comprehensive cancer network international prognostic index was more accurate at predicting progression-free survival than whole-body total MTV, while Cottereau et al. [26] demonstrated the opposite. Such studies are based on static PET acquisitions that measure radiopharmaceutical uptake heterogeneity only at a one time-point. However, the knowledge of regional heterogeneity in molecular features of cancer cells changes over time can have significant implications for tumor response to treatment and patient outcomes [27].

Alternatively, dynamic PET imaging, employed primarily in the research setting, can track PET radiopharmaceutical biodistribution in the body over time, offering dynamic analysis, including full kinetic modeling and potentially enhanced clinical tasks such as therapy response monitoring [28, 29]. As such, dynamic features derived from kinetic maps might contain additional information concerning the behavior of the tumor. Meanwhile, there have been only few published papers evaluating dynamic features due to the limitations of dynamic acquisition. In patients with non-small cell lung cancer (NSCLC), two studies investigated the correlation between dynamic and static radiomics features [30, 31]. Tixier et al. [30] analyzed static and parametric PET images with quantitative parameters (MTV, SUV_max_, SUV_mean_, heterogeneity) on 20 therapy-naive NSCLCs. They reported similar correlations and minor differences for metrics such as entropy and zone percentage quantifying intra-tumor uptake spatial distribution heterogeneity. However, they suggested further validation studies to compare the predictive or prognostic value of static versus parametric images for patient response or overall survival in NSCLC. Noortman et al. [31] evaluated a more extensive feature set (spatial intensity, shape, and texture radiomics features) derived from static and dynamic [^18^F]FDG PET of thirty-five NSCLC patients. They indicated that dynamic gray-level co-occurrence matrix (GLCM) features contain limited additional information compared to static radiomic features. However, the number of patients in the dataset was limited, and it is difficult to draw a general conclusion. This is noteworthy that the aforementioned studies [30, 31] have merely investigated dynamic features in lung cancer with no prediction of response to therapy; therefore, further investigation is needed to evaluate chemotherapy response prediction using dynamic features of lymphoma patients. Based on previous reports, certain dynamic features appear to offer more information than static features, which could lead to improved predictions. In the current study, we sought to investigate the performance of dynamic features derived from the dual-time-point (DTP) *K*_*i*_ to develop pre-therapy [^18^F]FDG PET/CT prediction models for response to chemotherapy in lymphoma patients.

## Materials and methods

**Fig. 1:**
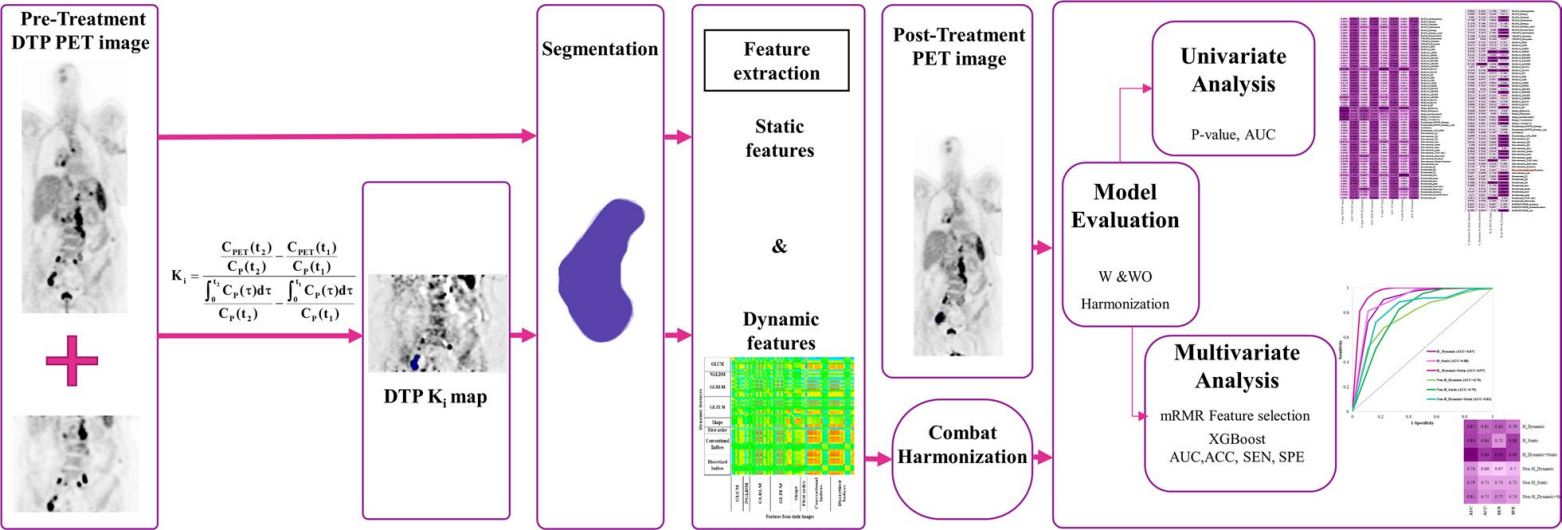
Five-step flowchart for the present study. (Step I) The K_i_ map was generated based on DTP imaging of pre-treatment PET data. (Step II) The SUV and *K*_*i*_ map were segmented to define VOI. (Step III) The LIFEx software was used to extract static and dynamic features. (Step IV) The ComBat harmonization was applied to each feature set to correct for the batch effect. A post-treatment PET scan was then used to assess the response to treatment. (Step V) prognostic models were developed to predict treatment outcomes for lymphoma patients and different classification metrics were reported for evaluation of models

Figure 1 summarizes the various steps involved in the study design. At first, the *K*_*i*_ map was generated from DTP imaging using pre-treatment PET data. Next, radiomics features were extracted from the regions of interest (ROIs) segmented from the SUV PET image and *K*_*i*_ map. Afterward, ComBat harmonization is applied to each feature set to adjust for the batch impact caused by the multi-center dataset. Next, the response to treatment was evaluated according to the post-treatment PET scan. Finally, predictive models are developed to predict the treatment response of lymphoma (Hodgkin and non-Hodgkin) patients.

### PET/CT imaging protocol and patient selection

**Fig. 2:**
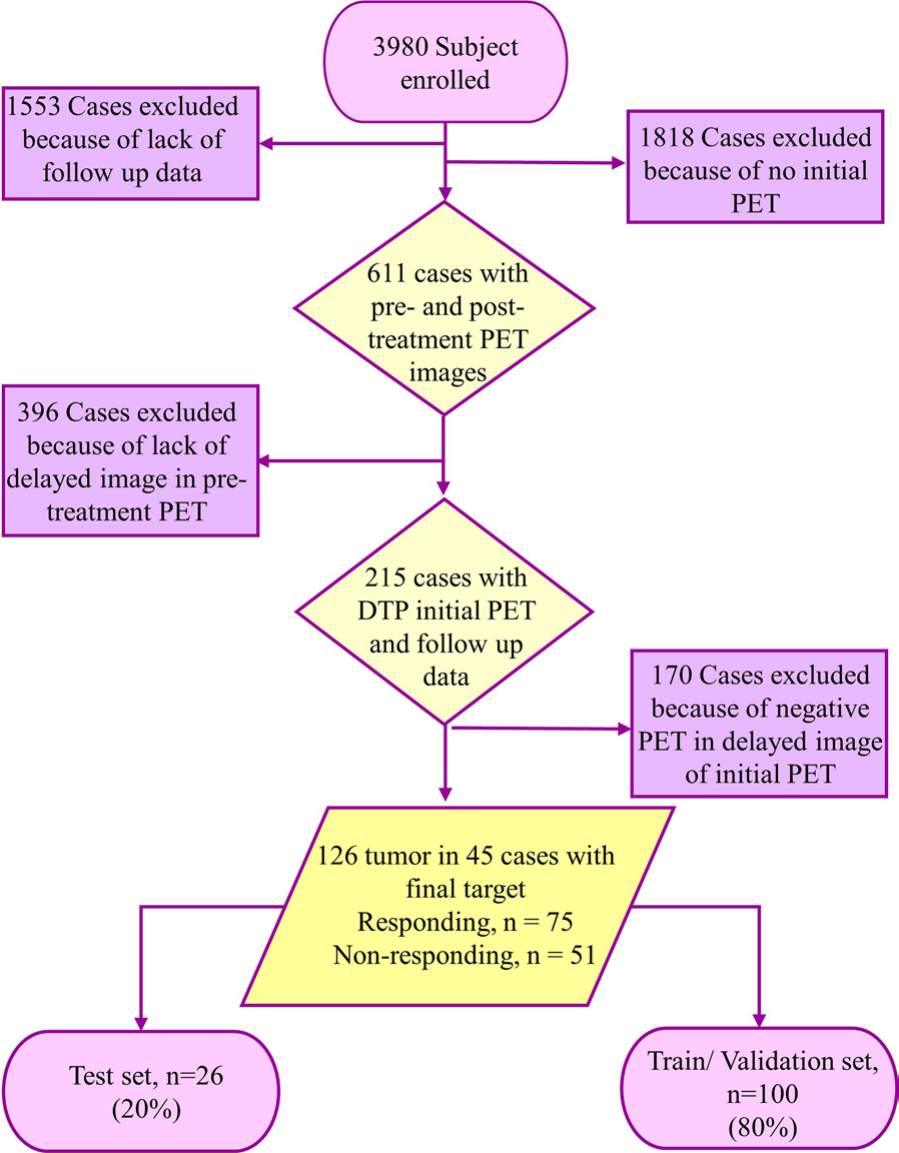
Inclusion and exclusion criteria followed in patient selection. A total of 126 lesion in 45 cases including 75 responding and 51 non-responding to treatment response were retained from an initial of 3980 patients. The criteria that were considered include: (1) patients have pre- and post-treatment PET/CT scans, (2) undergoing DTP PET image acquisition for initial PET scan, and (3) visible lesion in delayed image of pre-treatment PET

We searched for lymphoma patients with PET/CT scans from January 2013 until March 2022. We investigated around 4000 patients’ database records at two independent institutions, referred to as Centers 1 and 2. Medical records were carefully reviewed to identify which patients had pre- and post-treatment PET/CT scans, with the pre-treatment images acquired at DTP acquisition with a lesion in FOV of the delayed scan. The inclusion and exclusion criteria of patients are presented in Fig. 2. Overall, 26 patients from Center 1 and 19 from Center 2 were included.

All patients benefited from a second PET/CT evaluation after the first line of chemotherapy, specifically the doxorubicin (adriamycin), bleomycin, vinblastine, and dacarbazine (ABVD) regimen in Hodgkin lymphoma, and the rituximab, cyclophosphamide, doxorubicin, vincristine, and prednisone (R-CHOP) in non-Hodgkin lymphoma. Response to treatment was evaluated on a lesion basis according to Deauville criteria reported on the post-treatment PET scan [32]. A total of 126 lesions were individually classified as responding (*n* = 75) vs. non-responding (*n* = 51). The clinical characteristics of the patients are reported in Table 1. Before treatment, all patients underwent DTP [^18^F]FDG PET/CT scans with detailed key acquisition parameters of the datasets presented in Table 1.

**Table 1:** Summary of clinical characteristics of patients and image acquisition parameters in different centers

Variable	Center 1	Center 2
*Patient characteristic*		
Patient NO	26	19
Age(year) (Range)	36 (8–77)	38 (18–66)
Female	14	11
Male	12	8
*Histological type*		
Hodgkin	11	15
Non-Hodgkin	15	4
Lesion NO	81	45
*Lesion treatment response*		
Responding	55	20
Non-responding	26	25
*CT parameters*		
kVp (min, max, avg)	(80, 120, 119)	(80, 140, 120)
Tube current (min, max, avg)	(50, 250, 110)	(50, 150, 100)
Matrix size	(512 × 512)	(512 × 512)
Slice thickness (mm)	3	5
Pixel spacing (mm)	0.97	0.97
*PET parameters*		
Scanner	Biograph mCT	Biograph 6
PET acquisition time [min, max, avg (min)]	(47, 83, 61)	(47, 81, 64)
Delayed PET acquisition time [min, max, avg (min)]	(75, 228, 133)	(97, 220, 145)
Matrix size	200 × 200	168 × 168
Slice thickness (mm)	5	5
Pixel spacing (mm)	4.0728	4.0728
Reconstruction method	OSEM	OSEM
Iteration and subset	2i, 24 s	2i, 21 s
Filter Gauss FWHM (mm)	4	5
Frame duration (min)	3	3

### Generation of the *K*_*i*_ images

The image of the metabolic uptake rate was generated according to the DTP scan through an in-house MATLAB code [33, 34]. In short, the *K*_*i*_ map was defined as the slope of the Patlak equation from two time points, *t*_1_ (related to the routine static image data acquired 60-min post-injection) and *t*_2_ (the time of the delay scan) in the following Eq. (1):

1 $$K_{i} = \frac{{\frac{{C_{{{\text{PET}}}} \left( {t_{2} } \right)}}{{C_{{\text{P}}} \left( {t_{2} } \right)}} - \frac{{C_{{{\text{PET}}}} \left( {t_{1} } \right)}}{{C_{{\text{P}}} \left( {t_{1} } \right)}}}}{{\frac{{\int_{0}^{{t_{2} }} {C_{{\text{P}}} (\tau ){\text{d}}\tau } }}{{C_{{\text{P}}} \left( {t_{2} } \right)}} - \frac{{\int_{0}^{{t_{1} }} {C_{{\text{P}}} (\tau ){\text{d}}\tau } }}{{C_{{\text{P}}} \left( {t_{1} } \right)}}}}$$

where *C*_PET_(*t*) and *C*_P_(*t*) denote radiopharmaceutical concentrations at time *t* in tissue and plasma, ROIs, respectively. We derived a subject-specific input function for each patient by scaling a population-based input function described by Vriens et al. [35] to the patient’s image-derived blood pool activity derived from the routine static PET image. Spherical VOIs were manually delineated in the left ventricle and atrium at a sufficient distance from the myocardium, with 15 mm and 10 mm diameters, respectively. The VOIs were then averaged.

In most cases, the patient was taken off the bed following the whole-body (WB) PET prior to the delayed scan. As such, repositioning is a possible source of error for DTP evaluations. As a result, tumor-specific rigid registration between WB and delayed PET based on CT images was performed to maximize the accuracy of the *K*_*i*_ map.

### Image segmentation and feature extraction

**Fig. 3:**
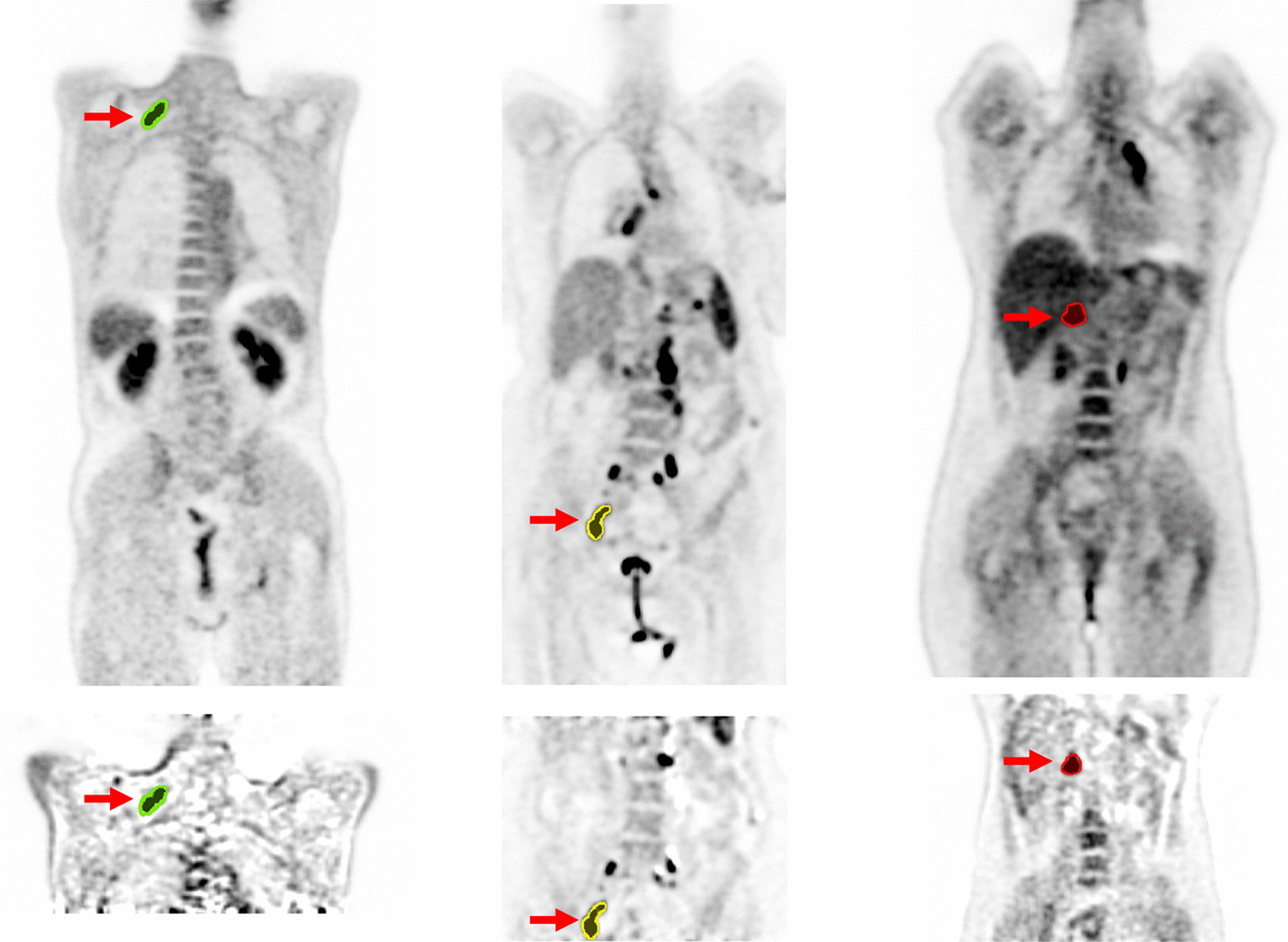
Examples of SUV (top) corresponding the DTP K_i_ (bottom) images showing segmented lesions

A threshold value of 30% of the maximum SUV was used to determine the VOI on the static images [36]. Then, the same VOI was manually delineated on the *K*_*i*_ images and modified by erasing or adding voxels to ensure the entire tumor was included in the VOI. Finally, all VOIs were reviewed by two nuclear medicine specialists. Figure 3 shows examples of segmented tumors on the parametric *K*_*i*_ and SUV images.

The LIFEx package (version 7.0.15) [37], which is standardized through the image biomarker standardization initiative (IBSI) [38], was used to extract radiomics features on PET images. First, all the *K*_*i*_ maps were multiplied by 100 to obtain the same scale as the SUV image. Then, the SUV and *K*_*i*_ images were processed using 64 bins, with the minimum and maximum image intensity values set to 0 and 20. Additionally, the voxel size was resampled to 4 × 4 × 4 mm^3^. A total of 65 radiomics features, including the category of gray-level co-occurrence matrix (GLCM, seven features), neighborhood gray-level different matrix (NGLDM, three features), gray-level run length matrix (GLRLM, eleven features), gray-level zone length matrix (GLZLM, eleven features), shape (five features), histogram (four features), conventional (twelve features), and discretized (twelve features) indices, were extracted for each lesion in both SUV and *K*_*i*_ images. Full details about the features are presented in Table 2.

**Table 2:** Radiomic features extracted from the SUV and *K*_*i*_ images

Category	Features
Gray-level co-occurrence matrix (GLCM)	Homogeneity, Energy, Contrast, Correlation, Entropy, Entropy log2, Dissimilarity
Neighboring gray-level dependence matrix (NGLDM)	Coarseness, Contrast, Busyness
Gray-level run length matrix (GLRLM)	SRE, LRE, LGRE, HGRE, SRLGE, SRHGE, LRLGE, LRHGE, GLNU, RLNU, RP
Gray-level zone length matrix (GLZLM)	SZE, LZE, LGZE, HGZE, SZLGE, SZHGE, LZLGE, LZHGE, GLNU, ZLNU, ZP
Shape	Sphericity, Compacity, Surface, Volume (ml, vx)
First-order features from the histogram	Entropy, Entropy log2, Uniformity, AUC_CSH
Conventional and discretized indices	Q1, Q2, Q3, min, mean, max, Standard Deviation, peak, TLG, Skewness, Kurtosis, Excess Kurtosis

### Harmonization

Harmonization was performed for all PET parameters using the ComBat harmonization method [39] to eliminate multicentre effects from radiomics features. In addition, ComBat harmonization removes batch effects based on an empirical Bayes framework using Bayes estimations for the location-scale parameters, including mean and variance for each variable [39–41].

### Univariate analysis

We calculated correlation coefficients between static and DTP features using Spearman’s rank method to identify features that might provide additional information. Receiver operating characteristic (ROC) curve analysis was used to assess the predictive power of each radiomics feature before and after the ComBat harmonization. The AUC of DTP and static features and the AUC of features before and after the ComBat harmonization were compared using Delong’s test. All the statistical analyses were performed in MedCalc (version 20.0.14; MedCalc Software Bvba). To assess the significance of the features, we also applied false discovery rate (FDR) Benjamini–Hochberg (BH) correction to correct for multiple comparisons, reporting *q* values. A *q* value of less than 0.05 defined statistical significance.

### Multivariate machine learning analysis

We developed various models using the DTP and static features before and after Combat harmonization. Our models were: (1) H_ DTP (harmonized radiomics features extracted from the DTP *K*_*i*_ map), (2) H_Static (harmonized features extracted from the SUV images), (3) H_ DTP + Static (combined harmonized features extracted from the DTP *K*_*i*_ map and the SUV images), (4) Non-H_ DTP (non-harmonized features extracted from the DTP *K*_*i*_ map), (5) Non-H_Static (non-harmonized harmonized features extracted from the SUV images), (6) Non-H_ DTP + Static (combined non-harmonized harmonized features extracted from the DTP *K*_*i*_ map and the SUV images).

First, we selected the most effective features by applying the minimum redundancy maximum relevance (mRMR) approach [42] to the input data. This algorithm selects a subset of features with maximum relevancy to the patient’s outcome and the most negligible correlation with each other simultaneously. Next, the classifiers were built with Python 3.7.4 and constructed using eXtreme Gradient Boosting (XGBoost version 1.6.1) machine learning algorithm [43]. XGBoost is an ensemble learning algorithm based on different decision trees. Finally, three different radiomic models based on the (1) static, (2) DTP, and (3) combination of DTP and static PET features were established to predict therapy response in lymphoma patients.

This study randomly divided the data into two groups: 80% for the model training and internal validation and 20% for the test. The test data were not used during model development. A subset of the training dataset was used to derive the models (80%), and the remainder (20%) was used for validation. We repeatedly trained a bootstrapped model with 1000 repetitions to find the optimal hyperparameters of models based on the random search method and AUC. Then, the optimal model was tested on the remaining 20% of the dataset (unseen during model training). This process was repeated 100 times to ensure the results were repeatable for different models. The mean ROC and the mean, standard deviation, and 95% confidence interval (CI) of AUC, accuracy (ACC), sensitivity (SEN), and specificity (SPE) were used to assess the predictive performance of the models. We used the Mann–Whitney test to determine significant differences between the models.

## Results

### Univariate analysis

**Fig. 4:**
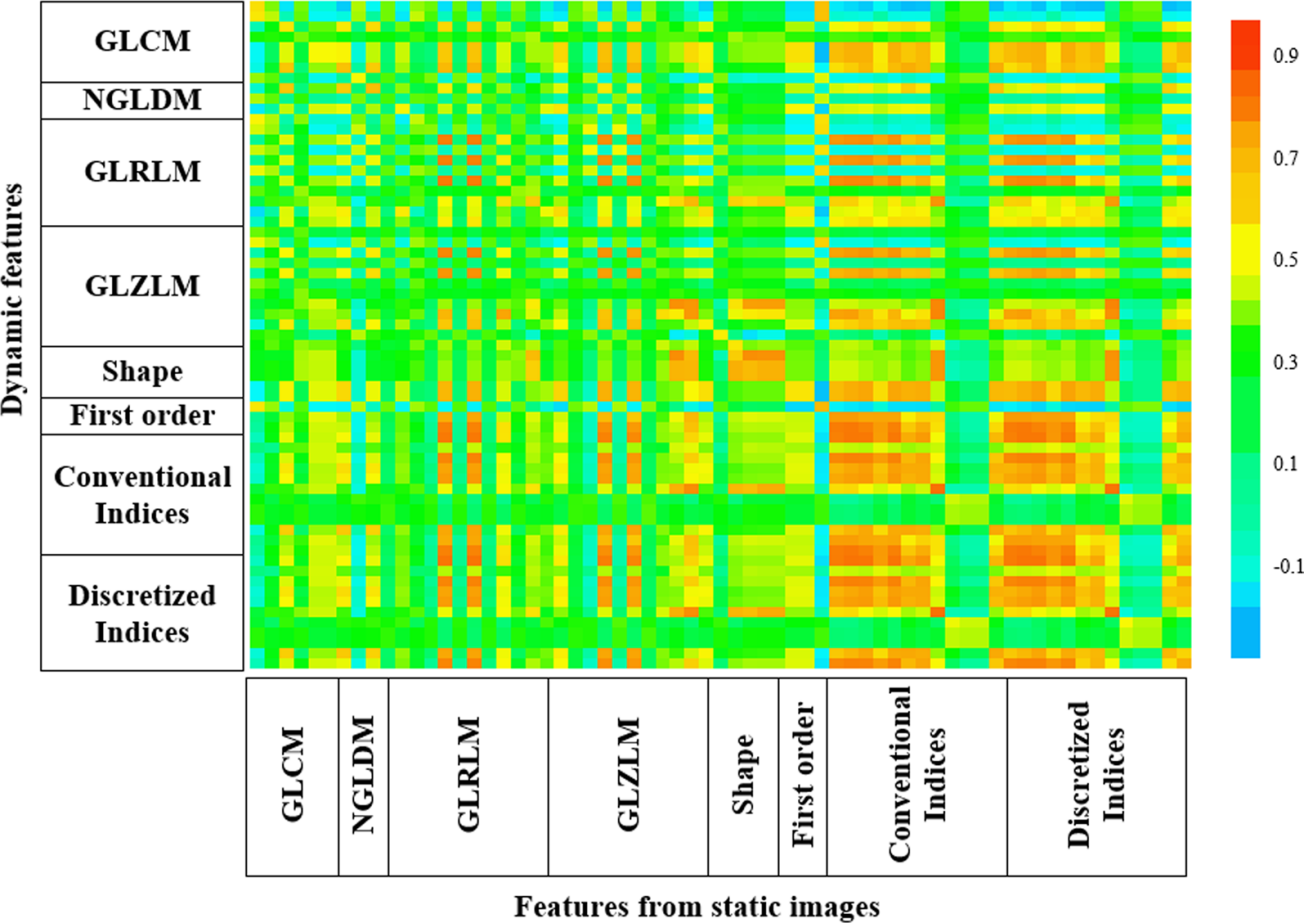
Spearman correlation matrix of dynamic and static features. Dynamic features with *ρ* < 0.7 contain additional information compared to static one

Spearman’s correlation matrix of static and DTP radiomics features is shown in Fig. 4. Using the Spearman’s correlation coefficient (*ρ*), the features with low (*ρ* < 0.5), moderate (0.5 < *ρ* < 0.7), and high (*ρ* > 0.7) correlation are reported in Table 3. DTP features with *ρ* < 0.7 contain additional information compared to static ones.

**Table 3:** Correlation of static and DTP features using Spearman’s correlation coefficient (*ρ*)

Low correlation *ρ* < 0.5, *p* value < 0.02	Moderate correlation 0.5 < *ρ* < 0.7, *p* value < 0.0001	High correlation *ρ* > 0.7, *p* value < 0.0001
GLCM_Correlation NGLDM_Busyness GLRLM_GLNU GLZLM_LZE GLZLM_SZLGE GLZLM_LZLGE GLZLM_LZHGE Conventional_Skewness Conventional_Excess Kurtosis Conventional_Kurtosis Discretized_Kurtosis Discretized_Excess Kurtosis	GLCM_Homogeneity GLCM_Energy GLCM_Contrast NGLDM_Coarseness NGLDM_Contrast GLRLM_SRE GLRLM_SRLGE GLRLM_LRE GLRLM_LGRE GLRLM_RP GLRLM_LRLGE GLZLM_SZE GLZLM_LGZE GLZLM_ZP Shape_Sphericity Shape_Compacity Conventional_min Discretized_min Discretized_Skewness Uniformity	GLCM_Entropy GLCM_Entropy_log2 GLCM_Dissimilarity GLRLM_HGRE GLRLM_SRHGE GLRLM_LRHGE GLRLM_RLNU GLZLM_HGZE GLZLM_SZHGE GLZLM_GLNU GLZLM_ZLNU Shape_Surface(mm2) Shape_Volume(mL) Shape_Volume(vx) Discretized_HISTO_Entropy Discretized_HISTO_Entropy_log2 Discretized_AUC_CSH Conventional_Q1 Conventional_Q2 Conventional_Q3 Conventional_mean Conventional_max Conventional_peak Conventional_TLG(mL) Conventional_std Discretized_Q1 Discretized_Q2 Discretized_Q3 Discretized_mean Discretized_max Discretized_peakSphere0.5 mL Discretized_TLG(mL) Discretized_std

The AUC, *p* value, and *q* value for each DTP and static feature before and after harmonization are reported in Additional file 1: Fig. S1. The significant differences in the ROC curves between DTP and static features, before and after harmonization, are compared using the Delong test and false discovery rate (FDR) *q* value (< 0.05) using the Benjamini–Hochberg procedure (BH), as shown in Additional file 1: Fig. S2. Table 4 shows the number of features whose performance (as AUC) significantly increased, decreased, or did not result in any difference before and after harmonization for both DTP and static features. No significant difference was observed among the ROC curves of DTP and static radiomics features. When comparing the ROC curves before and after harmonization, most of the harmonized features do not show any decreases or increases in performance against non-harmonized features.

**Table 4:** Results of the Delong test comparing AUCs of the DTP and static features with and without ComBat harmonization

Comparisons features	Significantly decreased	Significantly improved	No difference
Harmonized versus non-harmonized DTP	15	3	47
Harmonized versus non-harmonized Static	0	0	65
Harmonized static versus harmonized DTP	0	0	65
Non-harmonized static versus non-harmonized DTP	0	0	65

The number of features (out of 65) is shown as to having significantly lower, higher, or comparable performance

### Multivariate analysis

The mRMR algorithm selected ten from 65 features for static and DTP models. From a total of 20 features composed of 10 top DTP and static features, the combined static + DTP model used ten selected features applying the mRMR algorithm. All of the selected features for each model are presented in Table 5.

**Table 5:** Ten top features selected by mRMR algorithms for each model

H_DTP	H_Static	H_DTP + Static	Non-H_DTP	Non-H_Static	Non-H_DTP + Static
GLCM_Homogeneity	GLCM_Contrast	DTP GLCM_Energy	GLCM_Homogeneity	GLCM_Dissimilarity	DTP GLCM_Energy
GLCM_Energy	GLCM_Dissimilarity	DTP GLRLM_LRE	GLCM_Energy	GLRLM_SRE	DTP GLRLM_SRE
GLCM_Entropy_log2	GLRLM_SRHGE	DTP GLRLM_RP	GLCM_Entropy_log2	GLRLM_RLNU	DTP GLRLM_LRE
GLCM_Entropy	GLRLM_RLNU	DTP Uniformity	GLCM_Entropy	GLZLM_SZHGE	DTP GLRLM_RP
GLRLM_SRE	GLZLM_LZE	DTP Conventional_Skewness	GLRLM_SRE	Uniformity	DTP Shape_Surface
GLRLM_LRE	GLZLM_SZHGE	Static GLCM_Contrast	GLRLM_LRE	Conventional_std	DTP Conventional_Kurtosis
GLRLM_RP	Conventional_std	Static GLRLM_RLNU	GLRLM_RP	Conventional Skewness	DTP Uniformity
Shape_Surface	Conventional Skewness	Static GLZLM_LZE	SHAPE_Surface	Discretized_Q3	Static GLCM_Dissimilarity
Conventional Skewness	Discretized_std	Static Discretized_std	Conventional Kurtosis	Discretized_std	Static GLRLM_RLNU
Uniformity	Discretized Skewness	Static Discretized_Skewness	Uniformity	Discretized Skewness	Static Discretized_Skewness

**Fig. 5:**
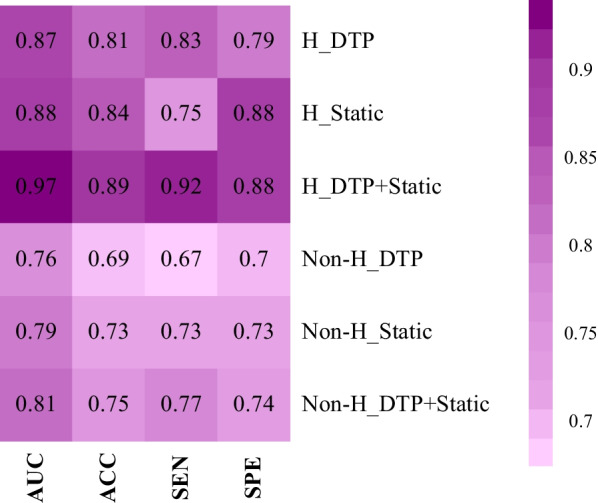
Heatmap of the performance of the DTP, static, and DTP + static models with and without ComBat harmonization; ACC: accuracy, AUC: area under the curve, SEN: sensitivity, SPE: specificity

**Fig. 6:**
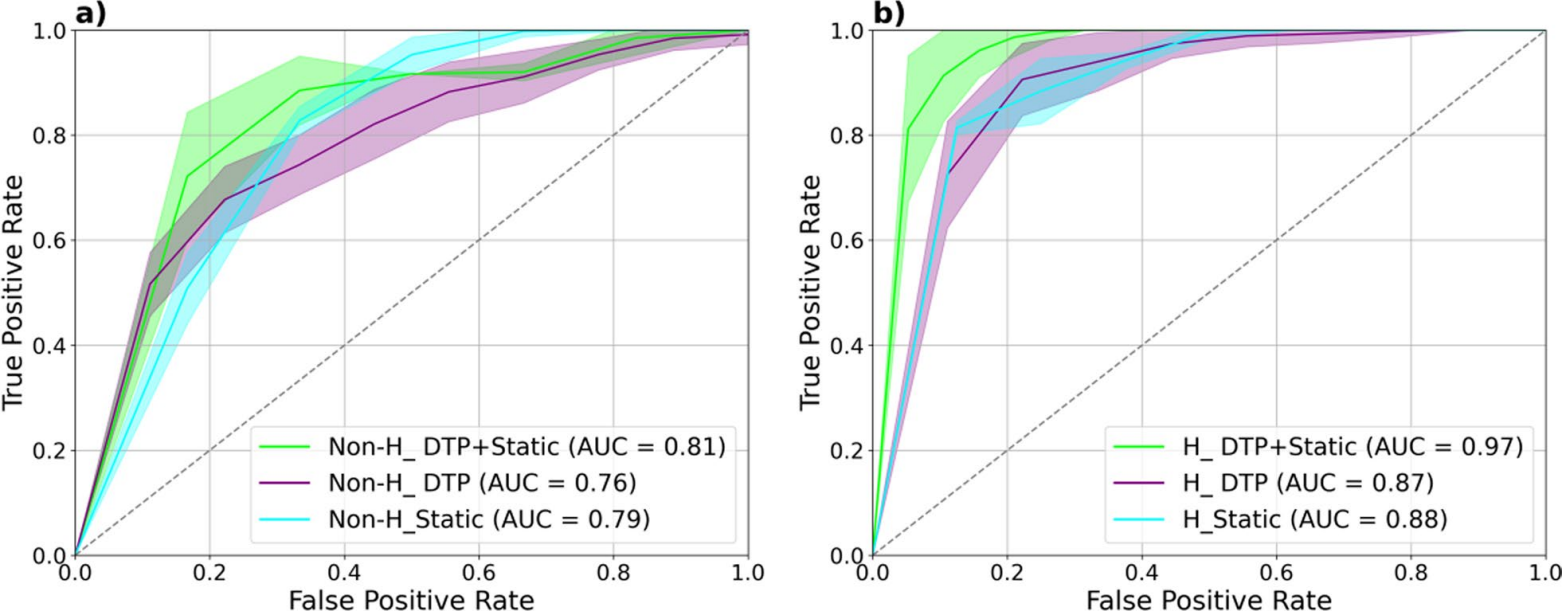
The ROC curves of the different models for prediction of response to therapy **a** before and **b** after ComBat harmonization. Solid lines are the mean ROC and the shaded regions represent one standard deviation around the average

**Fig. 7:**
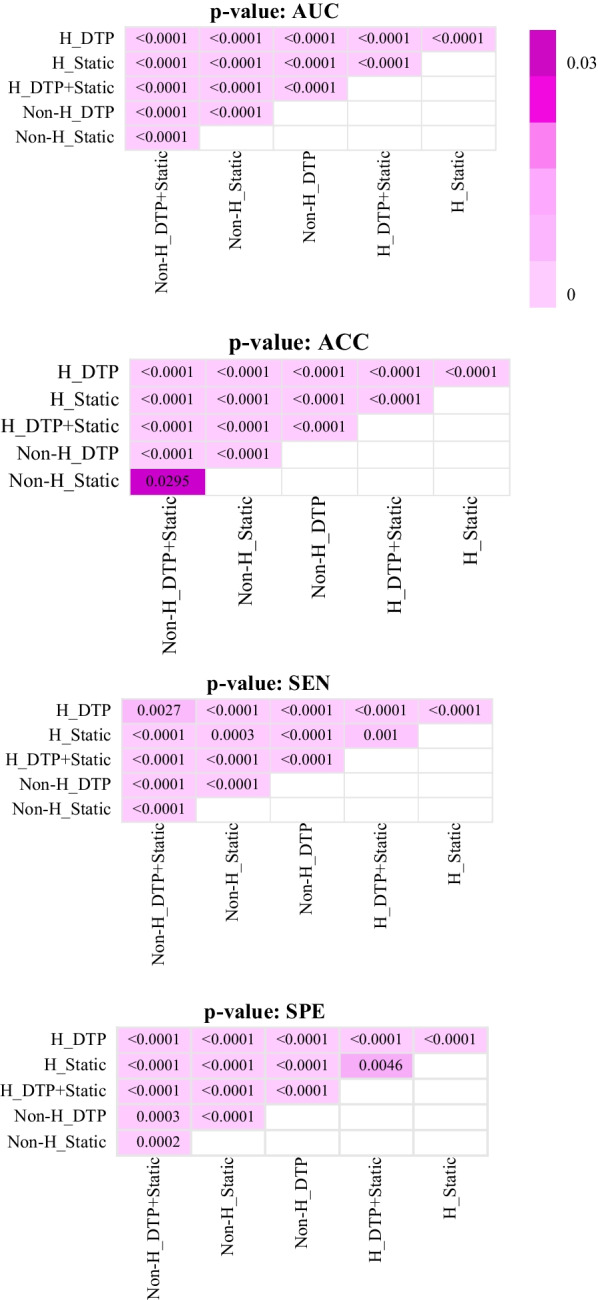
*p* Values for the comparison between the different models concerning the area under the curve (AUC), accuracy (ACC), sensitivity (SEN), and specificity (SPE)

The heat map of AUC, accuracy (ACC), sensitivity (SEN), and specificity (SPE) for different models, including DTP, static, and DTP + static, before and after harmonization to predict treatment response, are shown in Fig. 5. The confidence interval (CI) and mean and standard deviations (Mean ± STD) of AUC, ACC, SEN, and SPE for these models are summarized in Table 6. Figure 6 represents the ROC curve of these models for the test set. AUCs for all models have the highest values after harmonization. Before and after harmonization, the mean of AUC for the DTP model were 0.76 ± 0.02 and 0.87 ± 0.03, respectively. For static models, these values changed to 0.79 ± 0.02 and 0.88 ± 0.01, respectively, and for DTP + static model, these values were 0.81 ± 0.03 and 0.97 ± 0.02, respectively. Among the models, the combination of harmonized DTP and static features significantly improves the performance with AUC = 0.97 ± 0.02, ACC = 0.89 ± 0.05, SEN = 0.92 ± 0.09, SPE = 0.88 ± 0.05, respectively. The 95% CI for these parameters was 0.96–0.97, 0.88–0.90, 0.90–0.93, and 0.87–0.89, respectively. *p* Values are shown in Fig. 7, comparing models in terms of significant changes in AUC, ACC, SEN, and SPE. Majority of models had significant differences (*p* < 0.05).

**Table 6:** Mean, STD, and confidence interval (CI) of the area under the curve (AUC), accuracy (ACC), sensitivity (SNE), and specificity (SPE) in the test set for the different models studied

Different models	Mean ± STD (95% CI)			
	AUC	ACC	SEN	SPE
H_DTP	0.87 ± 0.03 (0.86–0.87)	0.81 ± 0.05 (0.80–0.82)	0.83 ± 0.08 (0.82–0.85)	0.79 ± 0.07 (0.78–0.80)
H_Static	0.88 ± 0.01 (0.87–0.88)	0.84 ± 0.03 (0.83–0.85)	0.75 ± 0.01 (0.74–0.75)	0.88 ± 0.05 (0.87–0.89)
H_DTP + Static	0.97 ± 0.02 (0.96–0.97)	0.89 ± 0.05 (0.88–0.90)	0.92 ± 0.09 (0.90–0.93)	0.88 ± 0.05 (0.87–0.89)
Non-H_DTP	0.76 ± 0.02 (0.75–0.76)	0.69 ± 0.04 (0.68–0.70)	0.67 ± 0.05 (0.66–0.68)	0.70 ± 0.06 (0.68–0.71)
Non-H_Static	0.79 ± 0.02 (0.78–0.79)	0.73 ± 0.02 (0.72–0.73)	0.73 ± 0.05 (0.72–0.74)	0.73 ± 0.03 0.72–0.74
Non-H_DTP + Static	0.81 ± 0.03 (0.81–0.82)	0.75 ± 0.05 (0.74–0.76)	0.77 ± 0.09 (0.75–0.79)	0.74 ± 0.06 (0.73–0.75)

## Discussion

Accurate prediction of response will improve treatment strategies and therefore optimize therapeutic results. In this study, we developed radiomics models for predicting the response of lesions to chemotherapy using the XGBoost classifier based on the static and DTP PET features selected by the mRMR algorithm in lymphoma patients. To this end, we extracted radiomics features from the SUV image and DTP K_i_ map, namely static and DTP features, respectively, and compared the predictive treatment response performance of DTP and static features. The present study investigated the potential information that DTP features may add to traditional features derived from the static PET images in 126 lesions of 45 lymphoma patients. Several studies have shown the significant potential of DTP imaging for generating parametric *K*_*i*_ images [33, 34, 44]. In the absence of list mode data, Van den Hoff et al. [34] proposed novel method to determine the metabolic uptake rate utilizing DTP images. Based on this study, we generate *K*_*i*_ map by determining the slope between the two time points. Only a few studies have investigated the performance of dynamic features. Tixier et al. [30] evaluated several parameters (SUV_max_, SUV_mean_, and MTV) and heterogeneity quantification in NSCLC. They reported high correlations for all parameters between SUV and parametric images, which indicates that heterogeneity quantification on parametric images does not offer additional information compared to static SUV images. However, in another study, Noortman et al. [31] found that certain dynamic GLCM radiomics features show different information than traditional radiomic in patients with NSCLC. In our study, 12 dynamic features contain additional information compared to static ones (see features with *ρ* < 0.5 in Table 3).

On the other hand, moderate correlation features provide a small amount of additional information (see features with 0.5 < *ρ* < 0.7 in Table 3). In agreement with Tixier et al. [30] and Noortman et al. [31] studies, most dynamic features show moderate and high correlations with static ones. Although the correlation of features found by the mentioned studies is not comparable to our results, the different types of lesion and acquisition protocols were investigated. We estimated the *K*_*i*_ map using the DTP method to achieve a simple and clinically feasible approach for deriving dynamic features. Several studies evaluated conventional PET metrics (SUV, MTV, and TLG) and showed the predicted value of treatment response in lymphoma patients [20–22, 45–48].

In addition, some studies investigated the role of PET radiomics features in predicting treatment response in lymphoma. Lue et al. [17] reported that wavelet HIR_GLRM_PET_ and RLNU_GLRM_CT_ are independent predictive factors for treatment response in patients with Hodgkin lymphoma. Tatsumi et al. [18] demonstrated that LGZE might help predict the treatment response of follicular lymphoma.

Univariate analysis of our study showed that some radiomics features might be predictive. For harmonized DTP features, the highest AUCs were achieved for GLCM_Energy, GLCM_Entropy, and uniformity (AUC = 0.73, *p* value = 0.0001, *q* value < 0.0005). Among static features, GLRLM_RLNU (AUC = 0.75, *p* value = 0.0001, *q* value = 0.0007) were found to be as most predictive features. Based on univariate results, there was no significant difference between the performance of most DTP and static radiomics features.

Specifically, several studies developed radiomic models for lymphoma patients to provide a prediction response to therapy. In a retrospective study included 57 bulky malignant lymphoma patients, Bouallègue et al. [23] presented a model incorporating static PET texture and shape features that achieved the highest predictive value with ROC AUC of 0.82 and 80% accuracy compared with other factors, including MTV and histology. Coskun et al. [19] developed the logistic regression model with cross-validation to predict treatment response using static PET features in DLBCL. They reported an accuracy of 0.87 and an AUC of 0.81. Finally, Jimenez et al. [15] proposed a radiomics model to predict ibrutinib response in lymphoma patients using static PET features trained by repeated cross-validation nested with the Gentle AdaBoost ensemble algorithm. They achieved an AUC of 0.86 (sensitivity, 92.9%, specificity, 81.4%; *p* < 0.001). Our study showed AUC = 0.88 for static features when taking advantage of the ComBat harmonization.

Since performing dynamic acquisition has limitations in clinical practice, the predictive value of dynamic features was not considered previously. We used the clinically feasible DTP PET imaging to achieve the *K*_*i*_ map. Our study sheds light on the possibility of treatment response prediction utilizing dynamic features by the DTP method. The results showed that DTP-feature yielded similar classification performance (AUC = 0.87) to static models (AUC = 0.88). Hence, since some DTP and static features had low and moderate correlations, they could serve as different markers. Previous studies reported improving performance by combining different markers, such as PET features and clinical data [9, 49]. Although it was out of the scope of the present investigation to add clinical data, we further took steps to build a novel model by combining DTP features with static ones. We found that this integrated model has the advantage of predicting treatment response with the highest AUC value (0.97). These results indicated that the H_DTP + Static model provided more accurate information and improved performance over other models we tried. Also, the performance of multivariate models was improved compared to univariate radiomics analysis. Due to the dual-centric nature of our study, we used ComBat harmonization to resolve the plausible batch effect. Univariate AUC of most DTP and static features did not differ significantly, and some of the features decreased before and after harmonization. However, as shown in Fig. 6, we observed higher AUCs and improvements in the predictive power of all multivariate models after harmonization, which were congruent with previous studies [50].

There were some limitations in this study. Foremost, the study cohort is relatively small; we used datasets from only two centers where external validation was lacking from different centers. However, we used the bootstrap technique to evaluate our models to address the limited sample size; further clinical studies are needed to verify our results with more extensive clinical databases. Moreover, obtaining full-time input function information for the standard Patlak method requires either arterial blood sampling or a long scan covering early time points of the blood pool. We used a scaled population-based input function for Patlak analysis to overcome this challenge, although the lack of ground truth information might have influenced the results. Another limitation of this study was the lack of multiple segmentations to assess the effect of segmentation variability on the extracted features. Finally, clinical data (patients' history and demographics, laboratory tests) were not considered in the model as the focus was on imaging features.

## Conclusion

Our results indicate the potential of combining dynamic and static features from FDG PET images to predict the treatment response in lymphoma patients. We used the dual-time-point framework to obtain the *K*_*i*_ maps and extract dynamic features, which can be applied in routine clinical practice. We demonstrated that the highest predictive performance of the XGBoost classifier with the mRMR algorithm was achieved when DTP and static features from FDG PET images were combined. We also demonstrated that ComBat harmonization significantly improved the performances of static, DTP, and combined static and DTP-based radiomics models toward significantly improved prediction of therapy response in lymphoma patients.

## Supplementary Information

**Additional file 1: Figure S1.** Shows the univariate AUC, *p*-values, and *q*-values heat map of DTP and static features with and without Combat harmonizations. **Figure S2.** Shows univariate Delong test *p*-values and *q*-values comparing the performance of combat harmonization in static and DTP features with and without Combat harmonization. Differences with *p* and *q* < 0.05 are considered statistically significant and highlighted in purple.

## Data Availability

The datasets used and analyzed during the current study are available from the corresponding authors on reasonable request.

## Abbreviations

ACC: Accuracy
AUC: Area under the curve
BH: Benjamini–Hochberg
CI: Confidence interval
DLBCL: Diffuse large B-cell lymphoma
DTP: Dual-time-point
DTP-feature: Features derived from dual-time-point
FDR: False discovery rate
IBSI: Image biomarker standardization initiative
mRMR: Minimum redundancy maximum relevance
MTV: Metabolic tumor volume
NSCLC: Non-small cell lung cancer
ROC: Receiver operating characteristic
ROIs: Regions of interest
SEN: Sensitivity
SPE: Specificity
STD: Standard deviations
SUV: Standardized uptake value
XGBoost: EXtreme Gradient Boosting

## References

[1] Wang H, Zhou Y, Li L, Hou W, Ma X, Tian R (2020). Current status and quality of radiomics studies in lymphoma: a systematic review. Eur Radiol.

[2] Acharya UR, Hagiwara Y, Sudarshan VK, Chan WY, Ng KH (2018). Towards precision medicine: from quantitative imaging to radiomics. J Zhejiang Univ-Sci B.

[3] Orlhac F, Nioche C, Klyuzhin I, Rahmim A, Buvat I (2021). Radiomics in PET imaging: a practical guide for newcomers. PET Clin.

[4] Hatt M, Tixier F, Pierce L, Kinahan PE, Le Rest CC, Visvikis D (2017). Characterization of PET/CT images using texture analysis: the past, the present… any future?. Eur J Nucl Med Mol Imaging.

[5] Lambin P, Rios-Velazquez E, Leijenaar R, Carvalho S, Van Stiphout RG, Granton P (2012). Radiomics: extracting more information from medical images using advanced feature analysis. Eur J Cancer.

[6] Cook GJ, Azad G, Owczarczyk K, Siddique M, Goh V (2018). Challenges and promises of PET radiomics. Int J Radiat Oncol* Biol* Phys.

[7] Yip SS, Aerts HJ (2016). Applications and limitations of radiomics. Phys Med Biol.

[8] Amini M, Nazari M, Shiri I, Hajianfar G, Deevband MR, Abdollahi H (2021). Multi-level multi-modality (PET and CT) fusion radiomics: prognostic modeling for non-small cell lung carcinoma. Phys Med Biol.

[9] Shiri I, Sorouri M, Geramifar P, Nazari M, Abdollahi M, Salimi Y (2021). Machine learning-based prognostic modeling using clinical data and quantitative radiomic features from chest CT images in COVID-19 patients. Comput Biol Med.

[10] Amini M, Hajianfar G, Avval AH, Nazari M, Deevband MR, Oveisi M (2022). Overall survival prognostic modelling of non-small cell lung cancer patients using positron emission tomography/computed tomography harmonised radiomics features: the quest for the optimal machine learning algorithm. Clin Oncol.

[11] Yousefirizi F, Decazes P, Amyar A, Ruan S, Saboury B, Rahmim A (2022). AI-based detection, classification and prediction/prognosis in medical imaging: towards radiophenomics. PET Clin.

[12] Hasani N, Paravastu SS, Farhadi F, Yousefirizi F, Morris MA, Rahmim A (2022). Artificial intelligence in lymphoma PET imaging: a scoping review (current trends and future directions). PET Clin.

[13] Lee JW, Lee SM (2018). Radiomics in oncological PET/CT: clinical applications. Nucl Med Mol Imaging.

[14] Cook GJ, Siddique M, Taylor BP, Yip C, Chicklore S, Goh V (2014). Radiomics in PET: principles and applications. Clin Transl Imaging.

[15] Jimenez JE, Dai D, Xu G, Zhao R, Li T, Pan T (2022). Lesion-based radiomics signature in pretherapy 18F-FDG PET predicts treatment response to ibrutinib in lymphoma. Clin Nucl Med.

[16] Parvez A, Tau N, Hussey D, Maganti M, Metser U (2018). 18F-FDG PET/CT metabolic tumor parameters and radiomics features in aggressive non-Hodgkin’s lymphoma as predictors of treatment outcome and survival. Ann Nucl Med.

[17] Lue K-H, Wu Y-F, Liu S-H, Hsieh T-C, Chuang K-S, Lin H-H (2020). Intratumor heterogeneity assessed by 18F-FDG PET/CT predicts treatment response and survival outcomes in patients with Hodgkin lymphoma. Acad Radiol.

[18] Tatsumi M, Isohashi K, Matsunaga K, Watabe T, Kato H, Kanakura Y (2019). Volumetric and texture analysis on FDG PET in evaluating and predicting treatment response and recurrence after chemotherapy in follicular lymphoma. Int J Clin Oncol.

[19] Coskun N, Okudan B, Uncu D, Kitapci MT (2021). Baseline 18F-FDG PET textural features as predictors of response to chemotherapy in diffuse large B-cell lymphoma. Nucl Med Commun.

[20] Sharma P, Gupta A, Patel C, Bakhshi S, Malhotra A, Kumar R (2012). Pediatric lymphoma: metabolic tumor burden as a quantitative index for treatment response evaluation. Ann Nucl Med.

[21] Tateishi U, Tatsumi M, Terauchi T, Ando K, Niitsu N, Kim WS (2015). Prognostic significance of metabolic tumor burden by positron emission tomography/computed tomography in patients with relapsed/refractory diffuse large B-cell lymphoma. Cancer Sci.

[22] Rogasch JM, Hundsdoerfer P, Hofheinz F, Wedel F, Schatka I, Amthauer H (2018). Pretherapeutic FDG-PET total metabolic tumor volume predicts response to induction therapy in pediatric Hodgkin’s lymphoma. BMC Cancer.

[23] Bouallègue FB, Al Tabaa Y, Kafrouni M, Cartron G, Vauchot F, Mariano-Goulart D (2017). Association between textural and morphological tumor indices on baseline PET-CT and early metabolic response on interim PET-CT in bulky malignant lymphomas. Med Phys.

[24] Sun Y, Qiao X, Jiang C, Liu S, Zhou Z (2020). Texture analysis improves the value of pretreatment 18F-FDG PET/CT in predicting interim response of primary gastrointestinal diffuse large B-cell lymphoma. Contrast Media Mol Imaging.

[25] Adams HJ, de Klerk JM, Fijnheer R, Heggelman BG, Dubois SV, Nievelstein RA (2015). Prognostic superiority of the National Comprehensive Cancer Network International Prognostic Index over pretreatment whole-body volumetric–metabolic FDG-PET/CT metrics in diffuse large B-cell lymphoma. Eur J Haematol.

[26] Cottereau A-S, Meignan M, Nioche C, Capobianco N, Clerc J, Chartier L (2021). Risk stratification in diffuse large B-cell lymphoma using lesion dissemination and metabolic tumor burden calculated from baseline PET/CT. Ann Oncol.

[27] Farhadi F, Rajagopal JR, Veziroglu EM, Abdollahi H, Shiri I, Nikpanah M (2023). Multi-scale temporal imaging: from micro-and meso-to macro-scale-time nuclear medicine. PET Clin.

[28] Kotasidis FA, Tsoumpas C, Rahmim A (2014). Advanced kinetic modelling strategies: towards adoption in clinical PET imaging. Clin Transl Imaging.

[29] Cheebsumon P, Velasquez LM, Hoekstra CJ, Hayes W, Kloet RW, Hoetjes NJ (2011). Measuring response to therapy using FDG PET: semi-quantitative and full kinetic analysis. Eur J Nucl Med Mol Imaging.

[30] Tixier F, Vriens D, Cheze-Le Rest C, Hatt M, Disselhorst JA, Oyen WJ (2016). Comparison of tumor uptake heterogeneity characterization between static and parametric 18F-FDG PET images in non-small cell lung cancer. J Nucl Med.

[31] Noortman WA, Vriens D, Slump CH, Bussink J, Meijer TW, de Geus-Oei L-F (2020). Adding the temporal domain to PET radiomic features. PLoS ONE.

[32] Barrington SF, Mikhaeel NG, Kostakoglu L, Meignan M, Hutchings M, Müeller SP (2014). Role of imaging in the staging and response assessment of lymphoma: consensus of the international conference on malignant lymphomas imaging working group. J Clin Oncol.

[33] Wu J, Liu H, Ye Q, Gallezot JD, Naganawa M, Miao T (2021). Generation of parametric *K*_*i*_ images for FDG PET using two 5-min scans. Med Phys.

[34] Van den Hoff J, Hofheinz F, Oehme L, Schramm G, Langner J, Beuthien-Baumann B (2013). Dual time point based quantification of metabolic uptake rates in 18 F-FDG PET. EJNMMI Res.

[35] Vriens D, de Geus-Oei L-F, Oyen WJ, Visser EP (2009). A curve-fitting approach to estimate the arterial plasma input function for the assessment of glucose metabolic rate and response to treatment. J Nucl Med.

[36] Oreiller V, Andrearczyk V, Jreige M, Boughdad S, Elhalawani H, Castelli J (2022). Head and neck tumor segmentation in PET/CT: the HECKTOR challenge. Med Image Anal.

[37] Nioche C, Orlhac F, Boughdad S, Reuzé S, Goya-Outi J, Robert C (2018). LIFEx: a freeware for radiomic feature calculation in multimodality imaging to accelerate advances in the characterization of tumor heterogeneity. Can Res.

[38] Zwanenburg A, Vallières M, Abdalah MA, Aerts HJ, Andrearczyk V, Apte A (2020). The image biomarker standardization initiative: standardized quantitative radiomics for high-throughput image-based phenotyping. Radiology.

[39] Johnson WE, Li C, Rabinovic A (2007). Adjusting batch effects in microarray expression data using empirical Bayes methods. Biostatistics.

[40] Fortin J-P, Parker D, Tunç B, Watanabe T, Elliott MA, Ruparel K (2017). Harmonization of multi-site diffusion tensor imaging data. Neuroimage.

[41] Fortin J-P, Cullen N, Sheline YI, Taylor WD, Aselcioglu I, Cook PA (2018). Harmonization of cortical thickness measurements across scanners and sites. Neuroimage.

[42] Peng H, Long F, Ding C (2005). Feature selection based on mutual information criteria of max-dependency, max-relevance, and min-redundancy. IEEE Trans Pattern Anal Mach Intell.

[43] Chen T, Guestrin C. Xgboost: a scalable tree boosting system. In: Proceedings of the 22nd ACM SIGKDD international conference on knowledge discovery and data mining; 2016. p. 785–94.

[44] Zhu W, Li Q, Bai B, Conti PS, Leahy RM (2014). Patlak image estimation from dual time-point list-mode PET data. IEEE Trans Med Imaging.

[45] Mettler J, Müller H, Voltin C-A, Baues C, Klaeser B, Moccia A (2019). Metabolic tumor volume for response prediction in advanced-stage Hodgkin lymphoma. J Nucl Med.

[46] Strati P, Ahmed MA, Fowler NH, Nastoupil LJ, Samaniego F, Fayad LE (2020). Pre-treatment maximum standardized uptake value predicts outcome after frontline therapy in patients with advanced stage follicular lymphoma. Haematologica.

[47] Albano D, Bosio G, Bianchetti N, Pagani C, Re A, Tucci A (2019). Prognostic role of baseline 18F-FDG PET/CT metabolic parameters in mantle cell lymphoma. Ann Nucl Med.

[48] Albano D, Bosio G, Pagani C, Re A, Tucci A, Giubbini R (2019). Prognostic role of baseline 18F-FDG PET/CT metabolic parameters in Burkitt lymphoma. Eur J Nucl Med Mol Imaging.

[49] Chen S-H, Wan Q-S, Zhou D, Wang T, Hu J, He Y-T (2019). A simple-to-use nomogram for predicting the survival of early hepatocellular carcinoma patients. Front Oncol.

[50] Shiri I, Amini M, Nazari M, Hajianfar G, Avval AH, Abdollahi H (2022). Impact of feature harmonization on radiogenomics analysis: prediction of EGFR and KRAS mutations from non-small cell lung cancer PET/CT images. Comput Biol Med.

