# Supplementary material for: Radiomics predictive modeling from dual-time-point FDG PET Ki parametric maps: application to chemotherapy response in lymphoma
Source: EJNMMI Res. 2023 Jul 26;13:70. doi: 10.1186/s13550-023-01022-0 (PMC10371962; doi:10.1186/s13550-023-01022-0)
Supplement: Supplementary file 1 — Additional file 1: Figure S1. Shows the univariate AUC, p-values, and q-values heat map of DTP and static features with and without Combat harmonizations. Figure S2. Shows univariate Delong test p-values and q-values comparing the performance of combat harmonization in static and DTP features with and without Combat harmonization. Differences with p and q < 0.05 are considered statistically significant and highlighted in purple. [file 13550_2023_1022_MOESM1_ESM.docx]

| 2E-04 | 1E-04 | 0.69 | 2E-04 | 1E-04 | 0.74 | 1E-03 | 4E-04 | 0.68 | 5E-04 | 1E-04 | 0.72 | GLCM_Homogeneity |
| --- | --- | --- | --- | --- | --- | --- | --- | --- | --- | --- | --- | --- |
| 3E-03 | 2E-03 | 0.66 | 2E-04 | 1E-04 | 0.75 | 0.01 | 0.01 | 0.64 | 5E-04 | 1E-04 | 0.73 | GLCM_Energy |
| 2E-04 | 1E-04 | 0.71 | 2E-04 | 1E-04 | 0.74 | 2E-03 | 5E-04 | 0.67 | 5E-04 | 1E-04 | 0.66 | GLCM_Contrast |
| 0.13 | 0.11 | 0.59 | 0.32 | 0.27 | 0.56 | 0.31 | 0.28 | 0.56 | 0.16 | 0.14 | 0.58 | GLCM_Correlation |
| 1E-03 | 7E-04 | 0.67 | 2E-04 | 1E-04 | 0.75 | 3E-03 | 1E-03 | 0.66 | 5E-04 | 1E-04 | 0.73 | GLCM_Entropy |
| 1E-03 | 7E-04 | 0.67 | 2E-04 | 1E-04 | 0.75 | 3E-03 | 1E-03 | 0.66 | 5E-04 | 1E-04 | 0.73 | GLCM_Entropy_log2 |
| 2E-04 | 1E-04 | 0.71 | 2E-04 | 1E-04 | 0.74 | 7E-04 | 1E-04 | 0.69 | 5E-04 | 1E-04 | 0.69 | GLCM_Dissimilarity |
| 0.01 | 3E-03 | 0.65 | 0.03 | 0.02 | 0.62 | 0.01 | 5E-03 | 0.64 | 0.07 | 0.05 | 0.60 | NGLDM_Coarseness |
| 2E-04 | 1E-04 | 0.72 | 2E-04 | 1E-04 | 0.70 | 9E-04 | 2E-04 | 0.68 | 0.01 | 0.01 | 0.64 | NGLDM_Contrast |
| 0.02 | 0.01 | 0.63 | 2E-03 | 1E-03 | 0.67 | 0.05 | 0.04 | 0.61 | 0.03 | 0.02 | 0.62 | NGLDM_Busyness |
| 4E-04 | 2E-04 | 0.69 | 2E-04 | 1E-04 | 0.72 | 2E-03 | 8E-04 | 0.67 | 9E-04 | 2E-04 | 0.69 | GLRLM_SRE |
| 3E-03 | 2E-03 | 0.66 | 2E-04 | 1E-04 | 0.71 | 0.01 | 0.01 | 0.64 | 2E-03 | 7E-04 | 0.68 | GLRLM_LRE |
| 0.06 | 0.04 | 0.60 | 0.01 | 4E-03 | 0.65 | 0.15 | 0.12 | 0.58 | 0.03 | 0.02 | 0.62 | GLRLM_LGRE |
| 2E-04 | 1E-04 | 0.70 | 2E-04 | 1E-04 | 0.71 | 2E-03 | 8E-04 | 0.66 | 0.03 | 0.02 | 0.62 | GLRLM_HGRE |
| 0.07 | 0.06 | 0.60 | 0.01 | 0.01 | 0.64 | 0.16 | 0.14 | 0.58 | 0.06 | 0.04 | 0.61 | GLRLM_SRLGE |
| 2E-04 | 1E-04 | 0.71 | 2E-04 | 1E-04 | 0.72 | 2E-03 | 8E-04 | 0.66 | 0.03 | 0.02 | 0.62 | GLRLM_SRHGE |
| 0.03 | 0.02 | 0.62 | 3E-03 | 2E-03 | 0.67 | 0.22 | 0.18 | 0.71 | 0.01 | 0.01 | 0.64 | GLRLM_LRLGE |
| 2E-04 | 1E-04 | 0.68 | 1E-03 | 8E-04 | 0.67 | 7E-04 | 1E-04 | 0.71 | 0.08 | 0.06 | 0.60 | GLRLM_LRHGE |
| 0.66 | 0.61 | 0.53 | 0.55 | 0.49 | 0.54 | 0.87 | 0.86 | 0.51 | 0.84 | 0.82 | 0.51 | GLRLM_GLNU |
| 2E-04 | 1E-04 | 0.70 | 6E-04 | 3E-04 | 0.68 | 7E-04 | 1E-04 | 0.75 | 5E-04 | 1E-04 | 0.71 | GLRLM_RLNU |
| 8E-04 | 4E-04 | 0.68 | 2E-04 | 1E-04 | 0.74 | 4E-03 | 2E-03 | 0.66 | 5E-04 | 1E-04 | 0.71 | GLRLM_RP |
| 2E-04 | 1E-04 | 0.69 | 0.01 | 0.01 | 0.64 | 2E-03 | 5E-04 | 0.67 | 0.03 | 0.02 | 0.62 | GLZLM_SZE |
| 5E-03 | 3E-03 | 0.65 | 2E-04 | 1E-04 | 0.69 | 0.01 | 0.01 | 0.64 | 0.02 | 0.01 | 0.63 | GLZLM_LZE |
| 0.02 | 0.01 | 0.63 | 7E-04 | 4E-04 | 0.68 | 0.05 | 0.04 | 0.61 | 4E-03 | 1E-03 | 0.66 | GLZLM_LGZE |
| 2E-04 | 1E-04 | 0.70 | 2E-04 | 1E-04 | 0.72 | 2E-03 | 1E-03 | 0.66 | 0.01 | 0.01 | 0.64 | GLZLM_HGZE |
| 3E-03 | 2E-03 | 0.66 | 0.12 | 0.09 | 0.60 | 0.02 | 0.01 | 0.62 | 0.16 | 0.13 | 0.58 | GLZLM_SZLGE |
| 2E-04 | 1E-04 | 0.70 | 2E-04 | 1E-04 | 0.71 | 2E-03 | 7E-04 | 0.67 | 0.01 | 3E-03 | 0.65 | GLZLM_SZHGE |
| 0.01 | 0.01 | 0.64 | 4E-04 | 2E-04 | 0.69 | 0.14 | 0.11 | 0.59 | 0.02 | 0.01 | 0.63 | GLZLM_LZLGE |
| 0.51 | 0.46 | 0.54 | 0.05 | 0.04 | 0.61 | 0.31 | 0.28 | 0.56 | 0.16 | 0.14 | 0.58 | GLZLM_LZHGE |
| 0.20 | 0.17 | 0.57 | 0.08 | 0.06 | 0.60 | 0.08 | 0.07 | 0.60 | 0.04 | 0.03 | 0.62 | GLZLM_GLNU |
| 2E-04 | 1E-04 | 0.70 | 2E-04 | 1E-04 | 0.70 | 7E-04 | 1E-04 | 0.71 | 1E-03 | 4E-04 | 0.67 | GLZLM_ZLNU |
| 4E-04 | 2E-04 | 0.68 | 2E-04 | 1E-04 | 0.70 | 3E-03 | 2E-03 | 0.66 | 0.01 | 4E-03 | 0.64 | GLZLM_ZP |
| 0.87 | 0.86 | 0.51 | 0.61 | 0.59 | 0.53 | 0.91 | 0.91 | 0.51 | 0.81 | 0.78 | 0.52 | Shape_Sphericity. |
| 0.96 | 0.96 | 0.50 | 0.72 | 0.72 | 0.52 | 0.59 | 0.57 | 0.53 | 0.24 | 0.21 | 0.57 | Shape_Compacity |
| 0.86 | 0.81 | 0.51 | 0.61 | 0.58 | 0.53 | 0.34 | 0.32 | 0.55 | 0.18 | 0.16 | 0.58 | Shape_Surface(mm2) |
| 0.87 | 0.85 | 0.51 | 0.61 | 0.59 | 0.52 | 0.29 | 0.26 | 0.56 | 0.15 | 0.12 | 0.58 | Shape_Volume(mL) |
| 0.87 | 0.85 | 0.51 | 0.61 | 0.59 | 0.52 | 0.29 | 0.26 | 0.56 | 0.15 | 0.12 | 0.58 | Shape_Volume(vx) |
| 2E-04 | 1E-04 | 0.70 | 2E-04 | 1E-04 | 0.75 | 9E-04 | 2E-04 | 0.68 | 5E-04 | 1E-04 | 0.72 | Discretized_HISTO_Entropy |
| 2E-04 | 1E-04 | 0.70 | 2E-04 | 1E-04 | 0.75 | 9E-04 | 2E-04 | 0.68 | 5E-04 | 1E-04 | 0.72 | Discretized_HISTO_Entropy_log2 |
| 2E-04 | 1E-04 | 0.72 | 2E-04 | 1E-04 | 0.75 | 7E-04 | 1E-04 | 0.71 | 5E-04 | 1E-04 | 0.73 | Uniformity |
| 4E-04 | 2E-04 | 0.62 | 2E-04 | 1E-04 | 0.70 | 7E-04 | 1E-04 | 0.66 | 0.01 | 0.01 | 0.64 | Discretized_AUC_CSH |
| 3E-03 | 2E-03 | 0.65 | 4E-03 | 3E-03 | 0.66 | 0.01 | 0.01 | 0.64 | 0.04 | 0.03 | 0.62 | Conventional_Q1 |
| 2E-04 | 1E-04 | 0.69 | 7E-04 | 4E-04 | 0.68 | 2E-03 | 7E-04 | 0.67 | 0.01 | 0.01 | 0.64 | Conventional_Q2 |
| 2E-04 | 1E-04 | 0.70 | 2E-04 | 1E-04 | 0.72 | 1E-03 | 4E-04 | 0.67 | 3E-03 | 9E-04 | 0.67 | Conventional_Q3 |
| 0.30 | 0.27 | 0.56 | 0.32 | 0.28 | 0.56 | 0.48 | 0.46 | 0.54 | 0.48 | 0.45 | 0.54 | Conventional_min |
| 2E-04 | 1E-04 | 0.68 | 2E-04 | 1E-04 | 0.70 | 2E-03 | 1E-03 | 0.66 | 0.01 | 0.01 | 0.64 | Conventional_mean |
| 2E-04 | 1E-04 | 0.70 | 2E-04 | 1E-04 | 0.72 | 7E-04 | 1E-04 | 0.68 | 2E-03 | 7E-04 | 0.67 | Conventional_max |
| 2E-04 | 1E-04 | 0.70 | 2E-04 | 1E-04 | 0.73 | 9E-04 | 2E-04 | 0.68 | 2E-03 | 6E-04 | 0.67 | Conventional_peak |
| 0.04 | 0.03 | 0.61 | 2E-03 | 1E-03 | 0.66 | 2E-03 | 6E-04 | 0.67 | 5E-04 | 1E-04 | 0.69 | Conventional_TLG (mL) |
| 0.05 | 0.04 | 0.61 | 0.61 | 0.55 | 0.53 | 0.03 | 0.02 | 0.62 | 0.45 | 0.41 | 0.55 | Conventional_Skewness |
| 0.09 | 0.08 | 0.59 | 0.22 | 0.18 | 0.57 | 0.08 | 0.07 | 0.60 | 0.16 | 0.14 | 0.58 | Conventional_Kurtosis |
| 0.09 | 0.08 | 0.59 | 0.22 | 0.18 | 0.57 | 0.08 | 0.07 | 0.60 | 0.16 | 0.14 | 0.58 | Conventional_Excess Kurtosis |
| 2E-04 | 1E-04 | 0.72 | 2E-04 | 1E-04 | 0.74 | 7E-04 | 1E-04 | 0.70 | 5E-04 | 1E-04 | 0.69 | Conventional_std |
| 4E-03 | 2E-03 | 0.65 | 0.01 | 0.01 | 0.63 | 0.01 | 0.01 | 0.63 | 0.07 | 0.05 | 0.60 | Discretized_Q1 |
| 2E-04 | 1E-04 | 0.68 | 4E-04 | 2E-04 | 0.69 | 2E-03 | 8E-04 | 0.66 | 0.07 | 0.05 | 0.60 | Discretized_Q2 |
| 2E-04 | 1E-04 | 0.70 | 2E-04 | 1E-04 | 0.72 | 1E-03 | 4E-04 | 0.67 | 5E-03 | 2E-03 | 0.66 | Discretized_Q3 |
| 0.28 | 0.24 | 0.56 | 0.32 | 0.28 | 0.55 | 0.44 | 0.42 | 0.54 | 0.95 | 0.95 | 0.50 | Discretized_min |
| 2E-04 | 1E-04 | 0.68 | 2E-04 | 1E-04 | 0.70 | 3E-03 | 1E-03 | 0.66 | 0.01 | 0.01 | 0.64 | Discretized_mean |
| 2E-04 | 1E-04 | 0.70 | 2E-04 | 1E-04 | 0.73 | 7E-04 | 1E-04 | 0.68 | 1E-03 | 4E-04 | 0.68 | Discretized_max |
| 2E-04 | 1E-04 | 0.70 | 2E-04 | 1E-04 | 0.73 | 9E-04 | 2E-04 | 0.68 | 2E-03 | 7E-04 | 0.67 | Discretized_peak |
| 0.05 | 0.03 | 0.61 | 4E-03 | 3E-03 | 0.65 | 2E-03 | 8E-04 | 0.67 | 1E-03 | 3E-04 | 0.68 | Discretized_TLG (mL) |
| 0.03 | 0.02 | 0.62 | 0.67 | 0.66 | 0.53 | 0.01 | 0.01 | 0.64 | 0.53 | 0.51 | 0.54 | Discretized_Skewness |
| 0.04 | 0.03 | 0.61 | 0.22 | 0.18 | 0.57 | 0.03 | 0.02 | 0.62 | 0.15 | 0.12 | 0.58 | DISCRETIZED_Kurtosis |
| 0.04 | 0.03 | 0.61 | 0.22 | 0.18 | 0.57 | 0.03 | 0.02 | 0.62 | 0.15 | 0.12 | 0.58 | DISCRETIZED_ExcessKurtosis |
| 2E-04 | 1E-04 | 0.71 | 2E-04 | 1E-04 | 0.74 | 7E-04 | 1E-04 | 0.70 | 5E-04 | 1E-04 | 0.69 | DISCRETIZED_std |
| q value NON-H_Static | P value NON-H_Static | AUC NON H_Static | q value NON-H_DTP | P value NON-H_DTP | AUC NON-H_DTP | q value H_Static | P value H_Static | AUC H_Static | q value H_DTP | P value H_DTP | AUC H_DTP |  |

**Fig S1** Univariate AUC, p-values and q-values heat map of DTP and static features with and without Combat harmonization

| 0.96 | 0.29 | 0.98 | 0.41 | 0.27 | 0.15 | 0.13 | 0.08 | GLCM_Homogeneity |
| --- | --- | --- | --- | --- | --- | --- | --- | --- |
| 0.96 | 0.07 | 0.98 | 0.09 | 0.31 | 0.24 | 0.24 | 0.22 | GLCM_Energy |
| 0.96 | 0.90 | 0.98 | 0.47 | 0.23 | 0.05 | 0.06 | 0.03 | GLCM_Contrast |
| 0.96 | 0.77 | 0.98 | 0.78 | 0.23 | 0.09 | 0.04 | 0.01 | GLCM_Correlation |
| 0.96 | 0.13 | 0.98 | 0.20 | 0.33 | 0.27 | 0.18 | 0.15 | GLCM_Entropy |
| 0.96 | 0.13 | 0.98 | 0.20 | 0.33 | 0.27 | 0.18 | 0.15 | GLCM_Entropy_log2 |
| 0.96 | 0.45 | 0.98 | 0.97 | 0.23 | 0.09 | 0.05 | 0.02 | GLCM_Dissimilarity |
| 0.96 | 0.57 | 0.98 | 0.47 | 0.30 | 0.20 | 0.04 | 0.01 | NGLDM_Coarseness |
| 0.96 | 0.73 | 0.98 | 0.43 | 0.23 | 0.05 | 0.06 | 0.02 | NGLDM_Contrast |
| 0.96 | 0.54 | 0.98 | 0.84 | 0.27 | 0.16 | 0.11 | 0.06 | NGLDM_Busyness |
| 0.96 | 0.53 | 0.98 | 0.71 | 0.37 | 0.31 | 0.17 | 0.13 | GLRLM_SRE |
| 0.96 | 0.35 | 0.98 | 0.54 | 0.43 | 0.37 | 0.18 | 0.13 | GLRLM_LRE |
| 0.96 | 0.33 | 0.98 | 0.42 | 0.31 | 0.20 | 0.14 | 0.10 | GLRLM_LGRE |
| 0.96 | 0.87 | 0.98 | 0.37 | 0.23 | 0.02 | 0.04 | 0.00 | GLRLM_HGRE |
| 0.96 | 0.42 | 0.98 | 0.56 | 0.31 | 0.24 | 0.10 | 0.05 | GLRLM_SRLGE |
| 0.96 | 0.79 | 0.98 | 0.42 | 0.23 | 0.04 | 0.04 | 0.00 | GLRLM_SRHGE |
| 0.96 | 0.32 | 0.98 | 0.22 | 0.13 | 0.00 | 0.31 | 0.31 | GLRLM_LRLGE |
| 0.96 | 0.74 | 0.98 | 0.04 | 0.32 | 0.26 | 0.04 | 0.01 | GLRLM_LRHGE |
| 0.96 | 0.87 | 0.98 | 0.98 | 0.88 | 0.86 | 0.18 | 0.15 | GLRLM_GLNU |
| 0.96 | 0.76 | 0.98 | 0.38 | 0.23 | 0.05 | 0.06 | 0.02 | GLRLM_RLNU |
| 0.96 | 0.28 | 0.98 | 0.37 | 0.31 | 0.22 | 0.15 | 0.11 | GLRLM_RP |
| 0.96 | 0.36 | 0.98 | 0.34 | 0.27 | 0.15 | 0.14 | 0.10 | GLZLM_SZE |
| 0.96 | 0.46 | 0.98 | 0.97 | 0.31 | 0.21 | 0.08 | 0.04 | GLZLM_LZE |
| 0.96 | 0.25 | 0.98 | 0.26 | 0.23 | 0.09 | 0.23 | 0.20 | GLZLM_LGZE |
| 0.96 | 0.59 | 0.98 | 0.71 | 0.23 | 0.05 | 0.04 | 0.00 | GLZLM_HGZE |
| 0.96 | 0.38 | 0.98 | 0.57 | 0.23 | 0.06 | 0.26 | 0.24 | GLZLM_SZLGE |
| 0.96 | 0.85 | 0.98 | 0.74 | 0.23 | 0.06 | 0.06 | 0.03 | GLZLM_SZHGE |
| 0.96 | 0.31 | 0.98 | 0.42 | 0.27 | 0.12 | 0.14 | 0.09 | GLZLM_LZLGE |
| 0.96 | 0.23 | 0.98 | 0.86 | 0.85 | 0.83 | 0.25 | 0.23 | GLZLM_LZHGE |
| 0.96 | 0.65 | 0.98 | 0.75 | 0.23 | 0.09 | 0.20 | 0.17 | GLZLM_GLNU |
| 0.99 | 0.98 | 0.98 | 0.46 | 0.31 | 0.23 | 0.14 | 0.09 | GLZLM_ZLNU |
| 0.96 | 0.72 | 0.98 | 0.81 | 0.23 | 0.07 | 0.06 | 0.03 | GLZLM_ZP |
| 0.96 | 0.83 | 0.98 | 0.88 | 0.97 | 0.97 | 0.27 | 0.26 | Shape_Sphericity. |
| 0.96 | 0.60 | 0.98 | 0.80 | 0.23 | 0.09 | 0.10 | 0.05 | Shape_Compacity |
| 0.96 | 0.86 | 0.98 | 0.67 | 0.73 | 0.69 | 0.05 | 0.02 | Shape_Surface(mm2) |
| 0.96 | 0.90 | 0.98 | 0.66 | 0.67 | 0.62 | 0.05 | 0.01 | Shape_Volume(mL) |
| 0.96 | 0.90 | 0.98 | 0.66 | 0.67 | 0.62 | 0.05 | 0.01 | Shape_Volume(vx) |
| 0.96 | 0.29 | 0.98 | 0.41 | 0.27 | 0.15 | 0.11 | 0.07 | Discretized_HISTO_Entropy |
| 0.96 | 0.29 | 0.98 | 0.41 | 0.27 | 0.15 | 0.11 | 0.07 | Discretized_HISTO_Entropy_log2 |
| 0.96 | 0.59 | 0.98 | 0.66 | 0.27 | 0.14 | 0.18 | 0.14 | Uniformity |
| 0.96 | 0.29 | 0.98 | 0.72 | 0.68 | 0.64 | 0.04 | 0.01 | Discretized_AUC_CSH |
| 0.99 | 0.97 | 0.98 | 0.68 | 0.23 | 0.08 | 0.04 | 0.01 | Conventional_Q1 |
| 0.96 | 0.88 | 0.98 | 0.55 | 0.23 | 0.07 | 0.05 | 0.02 | Conventional_Q2 |
| 0.96 | 0.60 | 0.98 | 0.92 | 0.23 | 0.05 | 0.04 | 0.01 | Conventional_Q3 |
| 0.99 | 0.99 | 0.98 | 0.98 | 0.23 | 0.07 | 0.11 | 0.06 | Conventional_min |
| 0.96 | 0.68 | 0.98 | 0.72 | 0.23 | 0.08 | 0.04 | 0.01 | Conventional_mean |
| 0.96 | 0.58 | 0.98 | 0.87 | 0.27 | 0.16 | 0.06 | 0.02 | Conventional_max |
| 0.96 | 0.53 | 0.98 | 0.90 | 0.27 | 0.12 | 0.05 | 0.01 | Conventional_peak |
| 0.96 | 0.32 | 0.98 | 0.64 | 0.16 | 0.01 | 0.11 | 0.07 | Conventional_TLG (mL) |
| 0.96 | 0.22 | 0.98 | 0.23 | 0.31 | 0.22 | 0.23 | 0.20 | Conventional_Skewness |
| 0.96 | 0.72 | 0.98 | 0.78 | 0.45 | 0.40 | 0.31 | 0.31 | Conventional_Kurtosis |
| 0.96 | 0.72 | 0.98 | 0.78 | 0.45 | 0.40 | 0.31 | 0.31 | Conventional_Excess Kurtosis |
| 0.96 | 0.63 | 0.98 | 0.89 | 0.27 | 0.14 | 0.07 | 0.03 | Conventional_std |
| 0.96 | 0.67 | 0.98 | 0.56 | 0.23 | 0.07 | 0.04 | 0.01 | Discretized_Q1 |
| 0.97 | 0.93 | 0.98 | 0.25 | 0.23 | 0.08 | 0.04 | 0.00 | Discretized_Q2 |
| 0.96 | 0.71 | 0.98 | 0.79 | 0.23 | 0.03 | 0.04 | 0.01 | Discretized_Q3 |
| 0.96 | 0.89 | 0.98 | 0.54 | 0.27 | 0.12 | 0.05 | 0.02 | Discretized_min |
| 0.96 | 0.71 | 0.98 | 0.72 | 0.23 | 0.06 | 0.04 | 0.01 | Discretized_mean |
| 0.96 | 0.54 | 0.98 | 0.92 | 0.27 | 0.16 | 0.07 | 0.03 | Discretized_max |
| 0.96 | 0.52 | 0.98 | 0.85 | 0.27 | 0.12 | 0.04 | 0.01 | Discretized_peak |
| 0.96 | 0.38 | 0.98 | 0.76 | 0.16 | 0.01 | 0.11 | 0.07 | Discretized_TLG (mL) |
| 0.96 | 0.08 | 0.98 | 0.08 | 0.29 | 0.18 | 0.24 | 0.22 | Discretized_Skewness |
| 0.96 | 0.23 | 0.98 | 0.54 | 0.31 | 0.23 | 0.15 | 0.11 | DISCRETIZED_Kurtosis |
| 0.96 | 0.23 | 0.98 | 0.54 | 0.31 | 0.23 | 0.15 | 0.11 | DISCRETIZED_ExcessKurtosis |
| 0.96 | 0.60 | 0.98 | 0.87 | 0.29 | 0.19 | 0.07 | 0.03 | DISCRETIZED_std |
| q-value Non-H_DTP & Static | p-value Non-H_DTP & Static | q-value H_DTP & Static | p-value H_DTP & Static | q-value W & WO H_Static | p-value W & WO H_Static | q-value W & WO H_DTP | p-value W & WO H_DTP |  |

**Fig S2** Univariate Delong test p-values and q-values comparing the performance of combat harmonization in static and DTP features with and without Combat harmonization. Differences with p and q < 0.05 are considered statistically significant and highlighted in purple
